# Prognostic Signatures Based on Thirteen Immune-Related Genes in Colorectal Cancer

**DOI:** 10.3389/fonc.2020.591739

**Published:** 2021-02-19

**Authors:** Xiao-Bo Ma, Yuan-Yuan Xu, Meng-Xuan Zhu, Lu Wang

**Affiliations:** ^1^ Department of General Surgery, The First Hospital of Shanxi Medical University, Taiyuan, China; ^2^ Department of Day Surgery Centre, The First Hospital of Shanxi Medical University, Taiyuan, China; ^3^ Department of Medical Oncology, Zhongshan Hospital, Fudan University, Shanghai, China; ^4^ Department of Plastic Surgery, Zhongshan Hospital, Fudan University, Shanghai, China

**Keywords:** colorectal cancer, WGCNA, immune, prognostic signature, LASSO

## Abstract

**Background:**

The immunosuppressive microenvironment is closely related to tumorigenesis and cancer development, including colorectal cancer (CRC). The aim of the current study was to identify new immune biomarkers for the diagnosis and treatment of CRC.

**Materials and Methods:**

CRC data were downloaded from the Gene Expression Omnibus and The Cancer Genome Atlas databases. Sequences of immune-related genes (IRGs) were obtained from the ImmPort and InnateDB databases. Gene set enrichment analysis (GSEA) and transcription factor regulation analysis were used to explore potential mechanisms. An immune-related classifier for CRC prognosis was conducted using weighted gene co-expression network analysis (WGCNA), Cox regression analysis, and least absolute shrinkage and selection operator (LASSO) analysis. ESTIMATE and CIBERSORT algorithms were used to explore the tumor microenvironment and immune infiltration in the high-risk CRC group and the low-risk CRC group.

**Results:**

By analyzing the IRGs that were significantly associated with CRC in the module, a set of 13 genes (CXCL1, F2RL1, LTB4R, GPR44, ANGPTL5, BMP5, RETNLB, MC1R, PPARGC1A, PRKDC, CEBPB, SYP, and GAB1) related to the prognosis of CRC were identified. An IRG-based prognostic signature that can be used as an independent potentially prognostic indicator was generated. The ROC curve analysis showed acceptable discrimination with AUCs of 0.68, 0.68, and 0.74 at 1-, 3-, and 5- year follow-up respectively. The predictive performance was validated in the train set. The potential mechanisms and functions of prognostic IRGs were analyzed, i.e., NOD-like receptor signaling, and transforming growth factor beta (TGFβ) signaling. Besides, the stromal score and immune score were significantly different in high-risk group and low-risk group (p=4.6982e-07, p=0.0107). Besides, the proportions of resting memory CD4^+^ T cells was significantly higher in the high-risk groups.

**Conclusions:**

The IRG-based classifier exhibited strong predictive capacity with regard to CRC. The survival difference between the high-risk and low-risk groups was associated with tumor microenvironment and immune infiltration of CRC. Innovative biomarkers for the prediction of CRC prognosis and response to immunological therapy were identified in the present study.

## Introduction

Colorectal cancer (CRC) is one of the most common malignant tumors, and its morbidity and mortality are on the rise worldwide. More than 1 million new cases of CRC are diagnosed globally every year (1), as are approximately 492,000 deaths (2). Although treatment techniques such as surgery, radiotherapy, and chemotherapy have been greatly improved, the prognosis remains poor. In the USA the respective 5-year survival rates of patients who underwent surgery to remove tumors for localized (stage I), regional (stages II and III), and distant (stage IV) CRC were 91.1%, 71.7%, and 13.3% (3). Most patients are at a progressive stage at the time of diagnosis and have thus missed the opportunity to undergo standard treatment, so more precise diagnoses and more effective treatments are urgently needed.

Currently the TNM classification system compiled up by the American Joint Committee on Cancer is the most robust prognostic indicator for stratifying patients (4). It is also used to guide clinical treatment for CRC. Because of tumor heterogeneity however, even patients at the same TNM stage may exhibit different survival times (5). Therefore, other auxiliary indicators are needed to predict prognoses more accurately, and provide an additional basis for therapy choices. Galon et al. (6) first reported that different subgroups of tumor-infiltrating lymphocytes could predict the prognosis of CRC patients in 2006. Numerous studies have subsequently revealed that tumor-infiltrating lymphocytes are closely related to the prognosis of CRC, and the degree of tumor regression after neoadjuvant radiotherapy in patients with locally advanced rectal cancer (7).

The main components of the tumor microenvironment include vascular cells, mesenchymal stem cells, tumor-associated fibroblasts, immune cells, inflammatory cells, and extracellular matrix, among others (8, 9). Most tumor cells express antigens recognized by host CD8^+^ T cells, but those that evade antitumor immune responses grow progressively (10). In the last decade immunotherapy-based drugs have been intensely investigated in cancer treatment, and immunotherapy has now become an effective therapy for several cancers (11, 12). In CRC immune checkpoint therapy can be effective in tumors that are mismatch-repair-deficient or have high levels of microsatellite instability, but ineffective in tumors that are mismatch-repair-proficient, microsatellite-stable, or have low levels of microsatellite instability (13). Therefore, characterizing the function of immunity in different responsive populations contributes to improving the efficacy of immunotherapy for CRC.

In the current study a CRC immune signature based on 13 prognostic immune-related genes (IRGs) was constructed, and its prognostic efficacy was verified using external validation datasets. The role of abnormal immune infiltration and tumor microenvironment heterogeneity in immunotherapy for CRC was also investigated.

## Materials and Methods

### Data Processing

The Cancer Genome Atlas (TCGA) CRC expression data, the corresponding phenotype, and survival data were downloaded from the xena database (http://xena.ucsc.edu/). The dataset contained a total of 434 samples, of which 383 were CRC samples and 51 were normal samples. The IRG dataset was downloaded from the ImmPort database (https://www.immport.org/shared/home) and the InnateDB database (https://www.innatedb.com/). After discarding repeated genes the ImmPort database contained 1,811 immune genes and the InnateDB database contained 1,226 immune genes. GSE72970 was downloaded from the Gene Expression Omnibus database to verify the efficacy of the survival prognosis model. GSE72970 contained 124 CRC disease samples. The limma package in R was used to conduct difference analysis on the expression profile data, and *p* < 0.05 and logFC > 1 as the threshold value were used to identify 4,793 differentially expressed genes (DEGs). A total of 569 IRGs were then identified *via* the intersection of the ImmPort and InnateDB immune databases and the DEGs.

### Functional Enrichment Analysis

Using DAVID (http://david.ncifcrf.gov/), the gene ontology function and CRC IRG pathways were enriched. Gene set enrichment analysis was used for functional analysis of candidate prognostic IRGs in key modules.

### Weighted Gene Co-Expression Network Analysis

The co-expression of IRGs in CRC was analyzed *via* weighted gene co-expression network analysis (WGCNA) using R software. A WGCNA algorithm was used to mine the gene modules that were synergistically expressed, then the correlation between those modules and the sample phenotype was analyzed to identify the modules that were most strongly related to the disease phenotype.

### Least Absolute Shrinkage and Selection Operator Analysis

Univariate Cox regression analysis was performed using the “Survival” package (https://CRAN.R-project.org/package=survival, Version:2.41-3), and 16 candidate IRGs associated with CRC prognosis were identified. Least absolute shrinkage and selection operator (LASSO) regression was conducted using the R package “glmnet” (14) to further screen the potential prognostic risk characteristics, and an immune-related CRC prognosis signature was generated. The risk score was then calculated as follows:

$$Risk\;score=\sum_{i=1}^{n}Coef\;i*Exp\;i$$

Coef is the regression coefficient and Exp is the expression value of the corresponding gene in each sample. CRC samples were divided into a high-risk group and a low-risk group based on the median risk score. The significance of the difference between the survival curves in the high-risk group and the low-risk group was tested *via* Kapan-Meier analysis. The area under the curve (AUC) was calculated to evaluate the predictive efficiency of the model in 1, 3, and 5 years. To verify whether the constructed risk predictor signature was an independent prognostic indicator, univariate analysis of clinical factors for CRC and multivariate Cox regression analysis of risk scores for CRC were performed. To test the predictive power of the prognostic model the GSE72970 dataset was downloaded from the Gene Expression Omnibus cohort to verify the predictive power of the prognostic model *via* Kaplan-Meier curve analysis and determine the 5-year area under the receiver operating characteristic (ROC) curve.

### Transcription Factor-mRNA Interaction Network Construction

Regulatory relationships between transcription factors and mRNA were downloaded from the TRRUST version 2 database (https://www.grnpedia.org/trrust/), and transcription factors with interactional relationships with the prognostic IRGs were screened out. A network incorporating transcription factors and IRGs was built using Cytoscape (15).

### Calculation of Immune Score and Matrix Score

Immune cells and stromal cells are two major types of non-tumor components in the tumor microenvironment, and it has been suggested that they are valuable in the diagnosis and prognosis of tumors. Gene expression characteristics of immune cells and stromal cells in the high-risk group and the low-risk group were calculated using the R package ESTIMATE.

### Assessment of Proportions of Immune Cell Types

CIBERSORT (http://cibersort.stanford.edu/) was used to characterize cell composition based on the gene expression profile of complex tissues. A characteristic white blood cell gene matrix (LM22) consisting of 547 genes was used to identify 22 immune cell types, including myeloid subsets, natural killer cells, plasma cells, naive and memory B cells, and T cells. CIBERSORT was combined with the LM22 eigenmatrix to estimate the proportions of 22 cell phenotypes in the high-risk group and the low-risk group. The ratio of all estimated immune cell types in each sample adds up to 1.

## Results

### WGCNA Identified Survival-Related Modules

**Figure 1:**
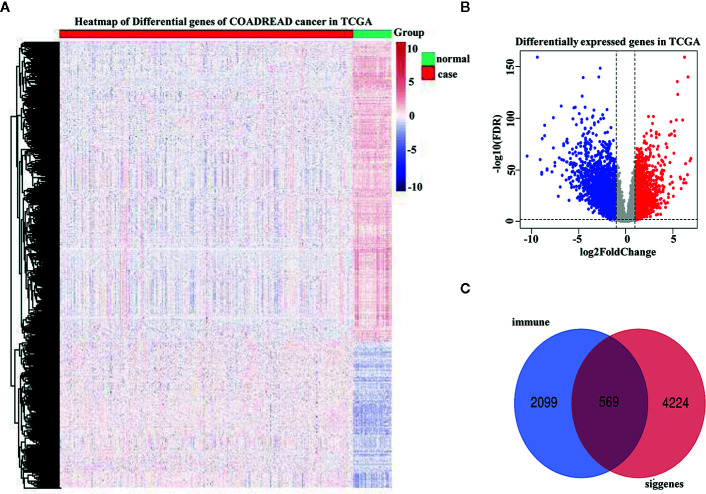
Differentially expressed genes were identified in The Cancer Genome Atlas (TCGA) analysis. **(A)** Heatmap of differentially expressed genes in TCGA analysis. **(B)** Distribution of upregulated and downregulated differentially expressed gene (DEG) in TCGA analysis. **(C)** Venn diagram depicting common immune-related genes shared by the TCGA dataset, ImmPort database, and InnateDB database.

**Figure 2:**
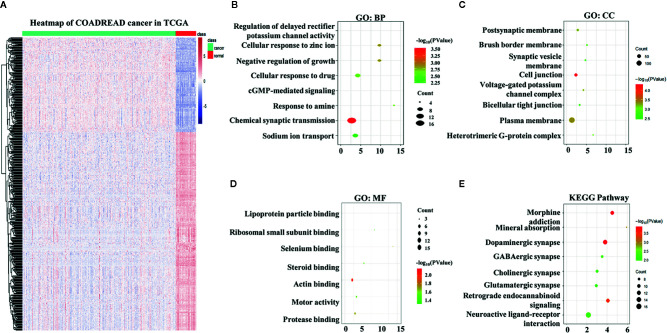
Overlapping immune-related genes (IRGs) and functional enrichment analysis. **(A)** Heatmap of IRGs in The Cancer Genome Atlas. **(B)** Biological process analysis of IRGs. **(C) **Cellular component analysis of IRGs. **(D)** Molecular function analysis of IRGs. **(E)** Kyoto Encyclopedia of Genes and Genomes analysis of IRGs.

A total of 4,793 differentially expressed genes were obtained *via* the limma package in R (**Figure 1A**), of which 1,574 were upregulated and 3,219 were downregulated (**Figure 1B**). The immune genes in the immune databases ImmPort and InnateDB were then merged, and 2,668 immune genes were identified. By intersecting the 2,668 immune genes and the 4,793 DEGs, 569 overlapping IRGs were identified (**Figure 1C**). A heat map of the IRGs is shown in **Figure 2A**. DAVID analysis indicated that the IRGs were mainly enriched in the Biological process (BP) like delayed rectifier potassium (**Figure 2B**), were mainly associated with the cellular component post (CC) like synaptic membrane (**Figure 2C**), were mainly included in molecular function (MF) like lipoprotein particle binding (**Figure 2D**). In Kyoto Encyclopedia of Genes and Genomes (KEGG) pathway analysis the IRGs were mainly enriched in the mineral absorption region, among others (**Figure 2E**).

**Figure 3:**
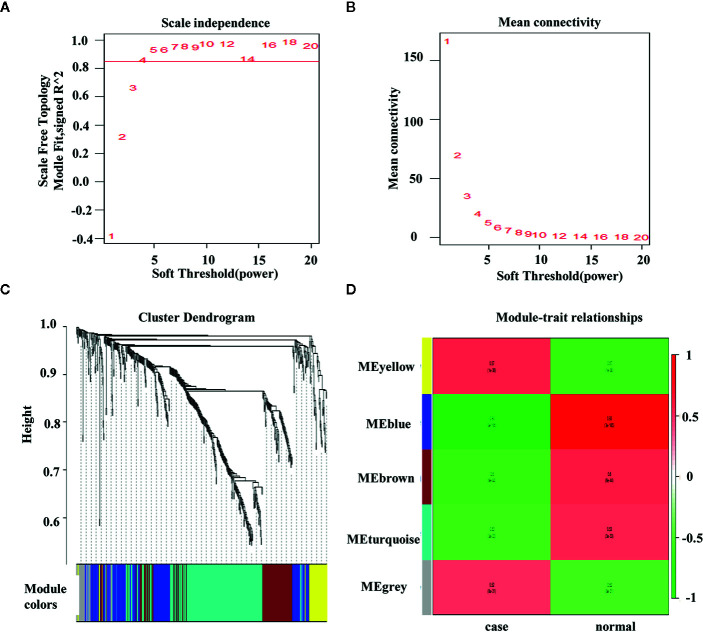
Weighted colorectal cancer gene co-expression network. **(A)** T scale−free fit index of various soft−thresholding powers. **(B)** Mean connectivity of various soft−thresholding powers. **(C)** A dendrogram of the differentially expressed genes clustered based on different metrics. **(D)** Heatmap of associations between module eigengenes and the progression of colorectal cancer. The association of the module and trait is calculated to be between −1 and 1.

In WGCNA, the optimal threshold value was 4 if the correlational coefficient was > 0.85 (**Figures 3A, B**). The genes were clustered *via* the average-linkage hierarchical clustering method, and five modules were obtained (**Figure 3C**). The blue module were negatively correlated with the disease (**Figure 3D**).

### Prognostic IRG Acquisition and Its Potential Functions

**Figure 4:**
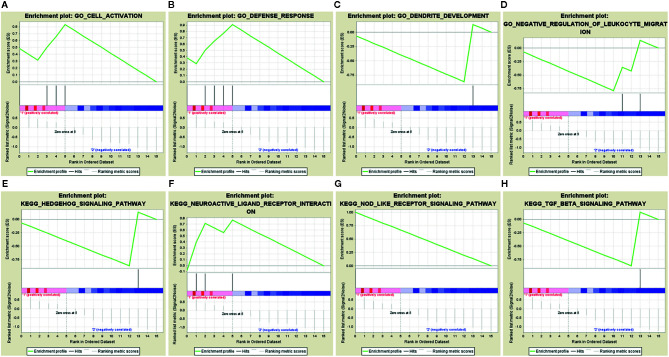
Gene set enrichment analysis of 16 prognostic immune-related genes. **(A–D)** GSEA in gene ontology (GO). **(E–H)** GSEA in KEGG pathways.

Sixteen survival-associated IRGs were acquired from the blue module (containing 124 IRGs) *via* univariate Cox regression (**Table 1**). In gene set enrichment analysis conducted to further investigate the possible roles of these genes with potential prognostic functions in gene ontology and KEGG pathways, the enriched biological functions identified included cell activation, defense responses, dendritic development, and negative regulation of leukocyte migration. The enriched KEGG pathways included the hedgehog pathway, neuroactive ligand receptor interaction, NOD-like receptor signaling, and transforming growth factor beta (TGFβ) signaling (**Figures 4A–H**).

**Table 1:** General characteristics of colorectal cancer‐specific immune‐related genes.

	Coefficient	*p*	HR	Lower df	Upper degree of freedom
CXCL1	-0.146	0.031	0.864	0.757	0.987
F2RL1	-0.441	0.008	0.643	0.464	0.892
CCL28	-0.153	0.026	0.858	0.750	0.982
LTB4R	0.192	0.044	1.212	1.005	1.460
GPR44	-0.230	0.013	0.795	0.662	0.954
ANGPTL5	0.526	0.001	1.692	1.226	2.336
BMP5	-0.180	0.001	0.835	0.751	0.929
RETNLB	-0.114	0.005	0.892	0.824	0.966
MC1R	0.341	0.002	1.406	1.135	1.740
PPARGC1A	-0.208	< 0.001	0.812	0.723	0.913
PRKDC	-0.383	0.013	0.682	0.504	0.923
CEBPB	0.447	0.014	1.564	1.096	2.233
SYP	0.179	0.028	1.196	1.019	1.402
LGALS4	-0.260	0.020	0.771	0.619	0.959
GAB1	-0.423	0.043	0.655	0.435	0.987
XDH	-0.120	0.037	0.887	0.792	0.993

### Prognostic IRG Transcriptional Regulatory Factors in CRC

**Figure 5:**
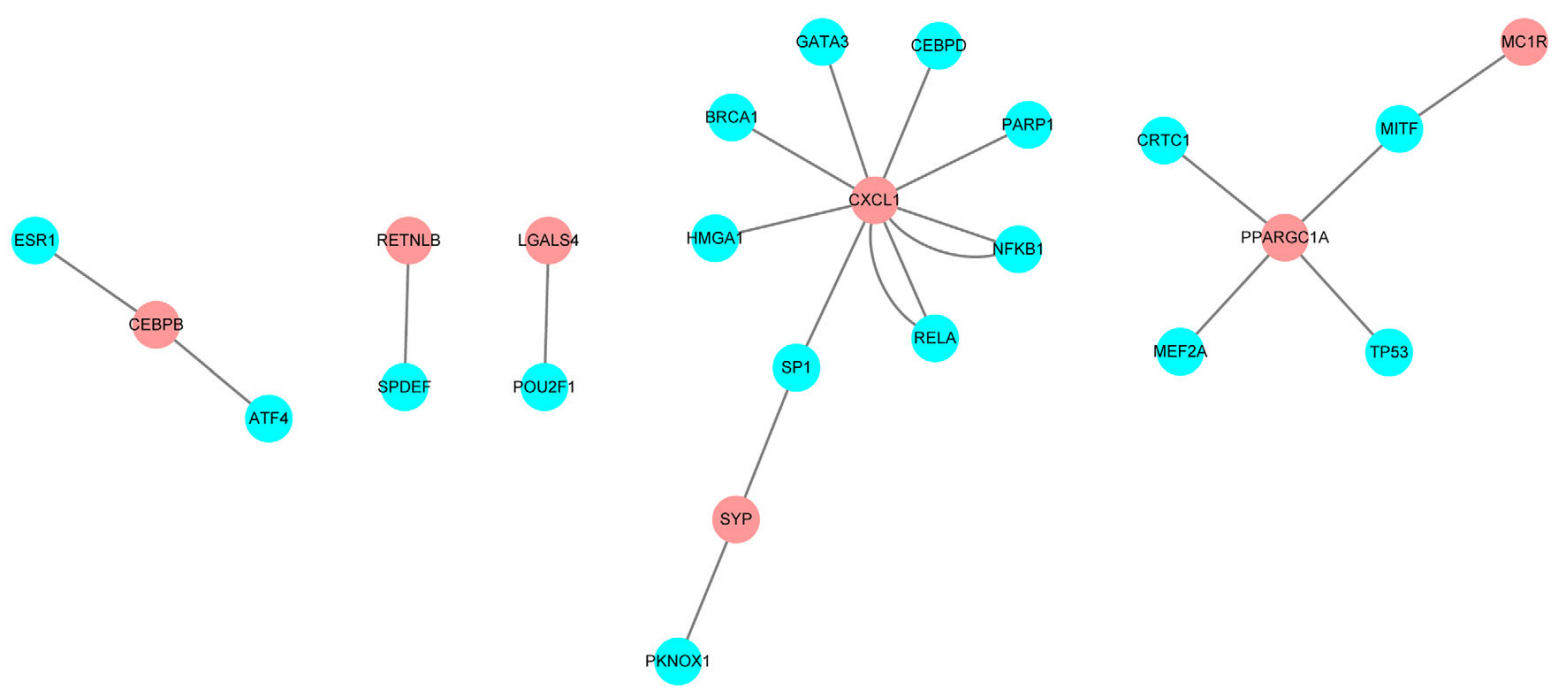
Regulatory network constructed based on clinically relevant transcription factors and immune-related genes.

Relationships between transcription factors and mRNA were downloaded from the TRRUST database, and screen out the transcription regulation factors which in relationship with the 16 candidate IRGs. A total of 21 interactional relationships were detected, and they involved the transcription factors TP53, GATA3, and breast-cancer susceptibility gene 1 (BRCA1) (**Figure 5**).

### Construction and Verification of Prognostic Classifier Based on IRGs

**Figure 6:**
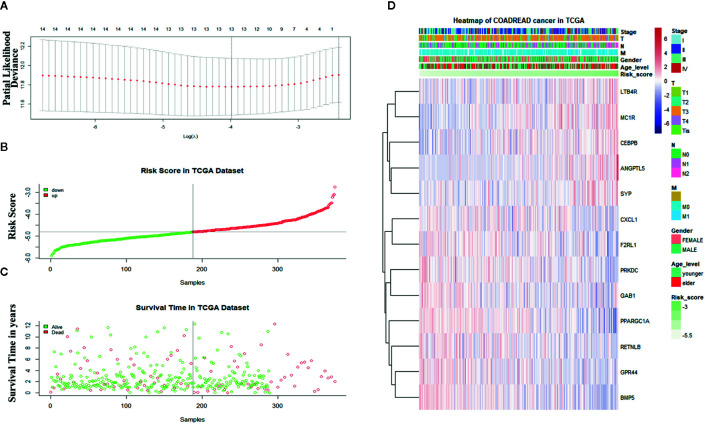
Construction of the immune-related gene-derived prognostic classifier. **(A)** Determination of the number of factors *via* least absolute shrinkage and selection operator analysis. **(B)** The survival duration and status of patients. **(C)** The distribution of risk score. **(D)** A heatmap of immune-related genes and the profile of each clinical feature in the classifier.

Sixteen prognostic IRGs were selected for LASSO regression analysis, and 13 genes were used to construct the prognostic classifier; C-X-C motif chemokine ligand 1 (CXCL1), F2R-like trypsin receptor 1 (F2RL1), leukotriene B4 receptor (LTB4R), GPR44, angiopoietin-like 5 (ANGPTL5), bone morphogenetic protein 5 (BMP5), resistin-like beta (RETNLB), melanocortin-1 receptor (MC1R), peroxisome proliferator-activated receptor γ coactivator 1α (PPARGC1A), protein kinase, DNA-activated, catalytic subunit (PRKDC), CCAAT enhancer binding protein beta (CEBPB), synaptophysin(SYP), and GRB2-associated-binding protein 1 (GAB1) (**Figure 6A**). The regression coefficient of each gene was calculated (**Table 2**). Risk scores were calculated based on regression coefficients obtained *via* the LASSO algorithm, and survival times in TCGA corresponding to risk scores were determined (**Figures 6B, C**). The median risk score was generated to separate the high-risk and low-risk groups. The risk group and the profile of each clinical feature are shown in **Figure 6D**.

**Table 2:** Immune-related genes in the prognostic classifier associated with overall survival in the gene set enrichment dataset.

	Univariate Cox regression analysis			LASSO coefficient
	Hazard ratio	95% confidence interval	*p*	
CXCL1	0.864	0.757–0.987	0.031	-0.055
F2RL1	0.643	0.464–0.892	0.008	-0.081
LTB4R	1.212	1.005–1.460	0.044	0.031
GPR44	0.795	0.662–0.954	0.013	-0.035
ANGPTL5	1.692	1.226–2.336	0.001	0.373
BMP5	0.835	0.751–0.929	0.001	-0.051
RETNLB	0.892	0.824–0.966	0.005	-0.022
MC1R	1.406	1.135–1.740	0.002	0.101
PPARGC1A	0.812	0.723–0.913	0.000	-0.141
PRKDC	0.682	0.504–0.923	0.013	-0.194
CEBPB	1.564	1.096–2.233	0.014	0.111
SYP	1.196	1.019–1.402	0.028	0.013
FGAB1	0.655	0.435–0.987	0.043	-0.183

LASSO, least absolute shrinkage and selection operator.

**Figure 7:**
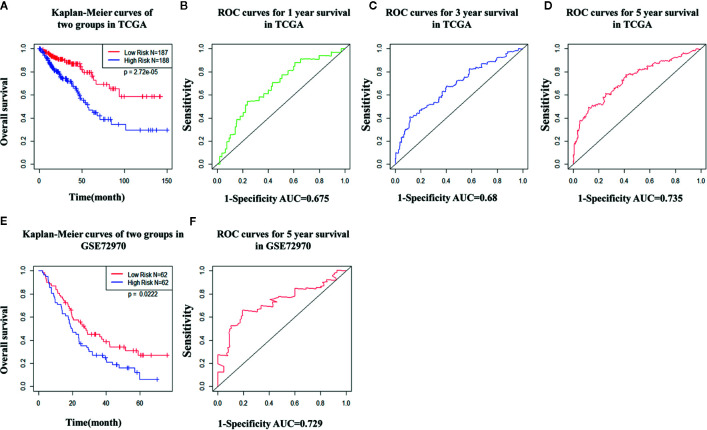
The distribution of time-dependent receiver operating characteristic (ROC) curves and Kaplan-Meier survival based on the integrated classifier in The Cancer Genome Atlas (TCGA) and gene set enrichment. **(A)** Kapan-Meier curve of the TCGA cohort. **(B–D)** ROC curves for 1-year, 3-year, and 5-year survival in the TCGA cohort. **(E)** Kapan-Meier curve of the gene set enrichment cohort. **(F)** ROC curve for 5-year survival in the gene set enrichment cohort. ROC, receiver operator characteristic; AUC, the area under the curve.

TCGA cohort patients with high risk scores exhibited a lower survival rate than those with low risk scores based on the Kaplan-Meier curve analysis (**Figure 7A**). In analysis of time-dependent ROC curves to assess the effects of the classifier the AUCs were 0.68 at 1 year, 0.68 at 3 years, and 0.74 at 5 years (**Figures 7B–D**). In GSE72970 analysis patients with high risk scores exhibited a lower survival rate than those with low risk scores (**Figure 7E**). The AUC was 0.729 at 5 years (**Figure 7F**). Univariate and multivariate Cox regression analysis revealed that age, TNM stage, and risk score in the prognosis model were significantly associated with survival (**Table 3**).

**Table 3:** Univariate and multivariate analyses of prognostic factors and overall survival of colorectal cancer patients in The Cancer Genome Atlas cohort.

	Univariate analysis			Multivariate analysis		
	HR	95% CI	*p*	HR	95% CI	*p*
Age	1.027	1.009–1.045	0.003	1.039	1.0154–1.062	0.001
Sex	0.797	0.518–1.227	0.302	–	–	–
Stage	1.940	1.493–2.520	<0.001	1.726	0.7418–4.016	0.205
M	3.507	2.074–5.929	<0.001	1.934	0.5769–6.485	0.285
N	1.805	1.398–2.331	<0.001	0.805	0.4703–1.378	0.429
T	2.725	1.740–4.268	<0.001	1.885	1.0143–3.504	0.045
Risk score	3.728	2.556–5.438	<0.001	3.738	2.2194–6.294	<0.001

### Stromal Scores and Immune Scores in the High-Risk and Low-Risk Groups

**Figure 8:**
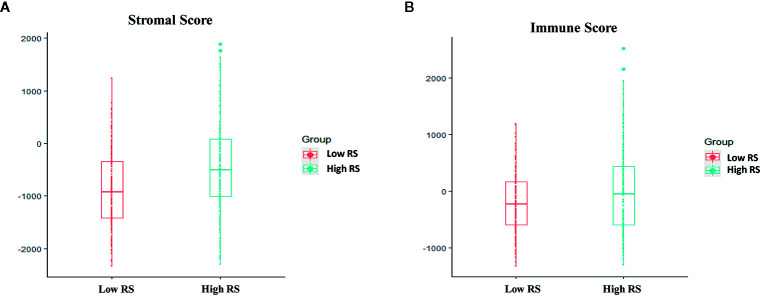
Associations between immune score, stromal score, and risk score. **(A)** Stromal scores of the high-risk group and the low-risk group in The Cancer Genome Atlas (TCGA) analysis. **(B)** Immune scores in the high-risk group and the low-risk group in TCGA analysis.

In GSE72970, stromal scores and the immune scores were significantly higher in the high-risk group than in the low-risk group (**Figure 8**).

### Leukocyte Subsets in the High-Risk and Low-Risk Groups

**Figure 9:**
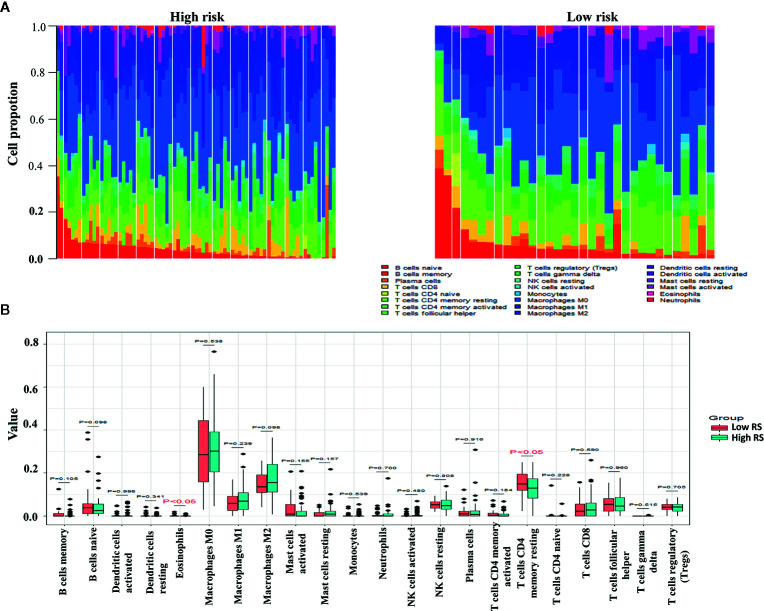
Differences between leukocyte subsets in the high-risk group and the low-risk group in The Cancer Genome Atlas (TCGA) cohort. **(A)** Mean proportions of 22 immune cells in the TCGA cohort. **(B)** Differential immune cell type expression was observed between the high-risk group and the low-risk group in the TCGA cohort.

**Figure 10:**
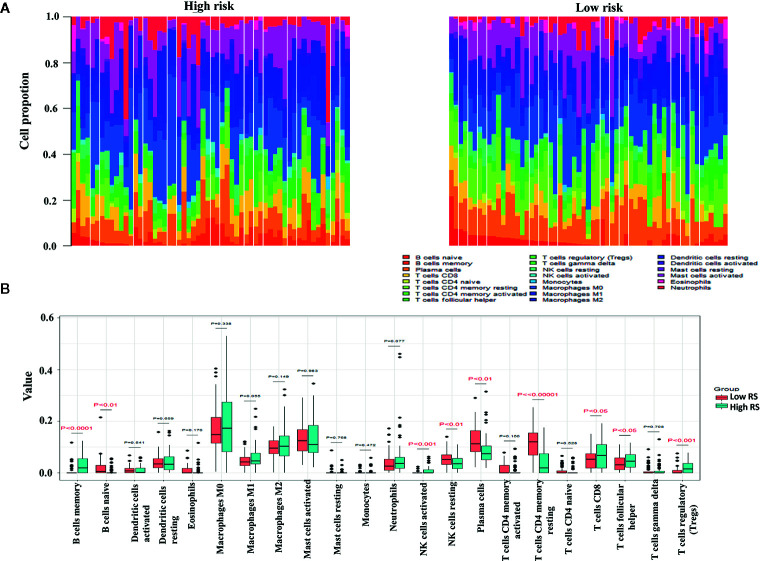
Differences in leukocyte cell subsets between the high-risk group and the low-risk group in the gene set enrichment (GSE) cohort. **(A)** Mean proportions of 22 immune cells in the GSE cohort. **(B)** Differential immune cell type expression was observed between the high-risk group and the low-risk group in the GSE cohort.

The proportions of resting memory CD4^+^ T cells and eosinophils differed significantly in the high-risk and low-risk groups (**Figures 9A, B**). In GSE72970 analysis the proportions of naive B cells, memory B cells, plasma cells, CD8^+^ T cells, CD4^+^ resting memory T cells, follicular helper T cells, regulatory T cells, resting natural killer cells, and activated natural killer cells differed significantly in the high-risk and low-risk groups (**Figures 10A, B**).

## Discussion

CRC is the third most common cancer in the world, with approximately 1.4 million cases diagnosed worldwide in 2012 (16). Remarkable progress has recently been made in two key areas at the immunology-cancer interface and microenvironment (17), and this may have substantial effects on future CRC diagnoses and treatments. In our study, we identified the prognostic signature based on the thirteen IRGs could categorize CRC patients into two subgroups with statistically different survival outcomes, which was validated in both TCGA and GSE72920 datasets. Additionally, we explored the underlying mechanisms using ESTIMATE and CIBERSORT analysis between risk groups.

Sixteen survival-associated IRGs were acquired from the key module and were significantly enriched in hedgehog signaling, NOD-like receptors and the TGFβ-signaling pathway. Aberrant hedgehog signaling in tumor cells can induce abnormal proliferation and invasion (18), and hedgehog signaling in the tumor microenvironment that targets cancer-associated fibroblasts can lead to angiogenesis (19), fibrosis (20), immune evasion (21), and neuropathic pain (22). Hedgehog-related genetic alterations mostly occur in basal cell carcinoma (85%) and sonic hedgehog-subgroup medulloblastoma (87%), and less frequently in breast cancer, CRC, and gastric cancer (23). NOD-like receptors are a relatively recent addition to the pattern recognition receptor superfamily (24). Increasing evidence suggests that chronic inflammation caused by aberrant NOD-like receptor signaling is a powerful driver of carcinogenesis, genetic mutation, tumor growth, and cancer progression (25). The TGFβ-signaling pathway is one of the important pathways in the tumorigenesis of CRC (26), and TGFβ activation in the tumor microenvironment can promote tumor-stromal interaction and lead to a malignant CRC phenotype and a poorer prognosis (27).

To investigate underlying molecular mechanisms, a transcription factor-mediated network was constructed to identify vital transcription factors that could regulate identified hub IRGs. TP53, GATA3, and BRCA1 were prominent in this network. TP53 can mediate several cellular stress responses such as DNA repair, cell-cycle arrest, and apoptosis, and suppress tumor formation (28). GATA3 is one of six members of the GATA family of transcription factors, and contains zinc-finger DNA binding domains that bind to 5′-(A/T) GATA (A/G)-3′ motifs (29). It regulates the specification and differentiation of various tissue types, and immunohistochemistry for GATA3 expression is primarily used in surgical pathology diagnosis for carcinomas originating from breast (30) or urothelial (31) tissue. BRCA1 and breast cancer 2 (BRCA2) are tumor suppressor genes that control aberrant cell proliferation and prevent tumor development (32). BRCA1 and/or BRCA2 mutation carriers are at a lifetime risk of developing breast cancer of up to 85%, and for ovarian cancer their lifetime risk is reportedly between 20% and 40% (33, 34).

In the present study the 13 IRGs that were strongly associated with CRC prognosis—CXCL1, F2RL1, LTB4R, GPR44, ANGPTL5, BMP5, RETNLB, MC1R, PPARGC1A, PRKDC, CEBPB, SYP, and GAB1—were used in the classifier investigation. Le Rollel et al. (35) reported that human CRC epithelia and myofibroblasts secrete elevated CXCL1 that facilitates blood vessel formation and recruitment of stromal and inflammatory cells, and promotes *in vivo* tumorigenic growth. There are two types of LTB4R; leukotriene B4 receptor 1 (BLT1) and leukotriene B4 receptor 2. BLT1 is a high-affinity LTB4R that is expressed by various subsets of leukocytes, and is responsible for LTB4-dependent leukocyte migration (36). BLT1 deficiency in Apc^min/+^ mice reportedly resulted in increased tumor size and increased numbers of intestinal tumors due to altered microbiota and increased chronic inflammation (37).

The tumor suppressor gene BMP5 has been investigated in myeloma, adrenocortical carcinoma, and breast cancer, and Chen et al. (38) reported that loss of BMP5 is an early event in CRC, and that low BMP5 expression was associated with recurrence and poorer prognoses. The intestinal goblet cell-specific protein RETNLB is markedly over-expressed in a human colon cancer cell line, and its expression is reportedly associated with histological grade of differentiation and lymph node metastasis in CRC patients (39). MC1R expression is associated with a higher risk of melanoma, and has been used as a target in melanoma therapy (40). In another bioinformatic study, MCR1 was one of the five immune genes used in the prognostic risk model of colon cancer (41). PPARGC1A 1α is a prominent regulator of mitochondrial biogenesis and metabolism (42). It has been reported that it can regulate cell proliferation and invasion *via* the AKT/GSK-3β/β-catenin pathway in human CRC SW620 and SW480 cells (43). PRKDC mediates DNA repair and maintains genomic stability, and it is reportedly upregulated in CRC cancerous tissues compared with normal tissues, and associated with chemoresistance (44). Wang et al. (45) reported that CEBPB is a critical effector of autophagy *via* regulation of autolysosome formation, and that forkhead box protein O1/CEBPB/nuclear factor kappa B signaling is required for C-C motif chemokine ligand 20 expression to augment chemoresistance in CRC. GAB1 belongs to the Grb2-associated binder family, which includes scaffolding adapter molecules that participate in transducing key signals from multiple receptors such as growth factors, cytokines, and antigen receptors (46). Bai et al. (47) identified GAB1 as a target of miR-409-3p in CRC, and demonstrated its unique function in CRC cell migration and invasion. In the current study, the area under the ROC curve confirmed the satisfactory predictive efficacy of the risk model based on the 13 prognosis-related IRGs identified, and the prognosis model risk score was an independent factor in multivariate Cox analysis. This innovative IRG-derived risk score model provides a new theoretical basis for predicting prognoses in CRC patients, and is expected to be applied in future clinical treatment.

The immune microenvironment affects the progression and prognosis of different cancers. The ESTIMATE algorithm was first presented by Yoshihara et al. (48) in 2013. ESTIMATE algorithm-derived immune scores were calculated in clear cell renal cell carcinoma, and higher immune scores, stromal scores, and ESTIMATE scores were associated with worse survival outcomes, advanced tumor grades and higher pathological stages (49). The same results were evident in patients with lower-grade glioma (50) and gastric cancer (51). In the current study immune scores were significantly higher in the high-risk group and were associated with shorter overall survival. Different degrees of risk may therefore be associated with differences in immune infiltration, and different patients may derive different benefits from immunotherapy.

The level of immune cell infiltration into the tumor is related to tumor growth, progression, and prognosis, and this has been a focus of research in recent years (52, 53). The biological software CIBERSORT developed in 2015 can calculate immune cell composition based on the gene expression profile of complex tissues (54). In the present study the expression profiles of CRC in the high-risk and low-risk groups were used to calculate immune cell compositions using CIBERSORT. In TCGA analysis CD4^+^ resting memory T cells were significantly higher in the low-risk group. CD4^+^ memory T cells impede the progression of tumor cells by supporting the proliferation of CD8^+^ cells, which move to tumor-related tissues and differentiate into effector cells. In one study increased disease-free survival was directly associated with higher proportions of resting and activated CD4^+^ memory T cells in breast cancer, implying an anti-tumor role of CD4^+^ memory T cells (55).In gene set enrichment analysis there were higher proportions of memory B cells, activated natural killer cells, CD8^+^ T cells, follicular helper T cells, and regulatory T cells in the high-risk group, and comparatively larger fractions of naive B cells, resting natural killer cells, CD4^+^ resting memory T cells, and plasma cells in the low-risk group. Lohr et al. (56) reported that mature plasma cells in tumor tissues were associated with a better prognosis in small cell lung cancer. Flammiger et al. (57) reported that Prostate-specific antigen recurrence‐free survival was lower in patients with higher densities of FOXP3^+^ regulatory T cells, and that high levels of FOXP3^+^ regulatory T cells were associated with advanced prostate cancer tumor stage. We conclude that to an extent differences in immune infiltration may explain the differences in prognoses in high-risk and low-risk patients. The limitation of our study is that the cohort did not consisted of patients who treated with immune checkpoint inhibitors, therefore, although we find the potential immune IRGs but it’s not clear if their classification using the immune-related genes are useful for predicting IO therapy in CRC.

In conclusion, in the current study an immune risk score model for CRC was established that could provide effective survival predictions in patients with CRC. Risk score was also significantly associated with immune score, stromal score, and immune cell infiltration. The study generated an alternative tool for survival prediction and treatment guidance in CRC.

## Data Availability Statement

The original contributions presented in the study are included in the article/supplementary material. Further inquiries can be directed to the corresponding authors.

## Author Contributions

X-BM designed the study, conducted the experimental process and literature search, and generated the figures. Y-YX, M-XZ, and LW wrote and edited the manuscript. All authors contributed to the article and approved the submitted version.

## Conflict of Interest

The authors declare that the research was conducted in the absence of any commercial or financial relationships that could be construed as a potential conflict of interest.

The reviewer P-FZ declared a shared affiliation, with no collaboration, with several of the authors, M-XZ, LW, to the handling editor at the time of review.

## Abbreviations

ANGPTL5, angiopoietin-like 5; AUC, area under the curve; BLT1, leukotriene B4 receptor 1; BMP5, bone morphogenetic protein 5; BRCA1, breast cancer 1; BRCA2, breast cancer 2; CEBPB, enhancer binding protein beta; CRC, colorectal cancer; CXCL1, C-X-C motif chemokine ligand 1; F2RL1, F2R-like trypsin receptor 1; GAB1, GRB2-associated-binding protein 1; IRG, immune-related gene; KEGG, Kyoto Encyclopedia of Genes and Genomes; LASSO, least absolute shrinkage and selection operator; LTB4R, leukotriene B4 receptor; MC1R, melanocortin-1 receptor; OS, overall survival; PPARGC1A 1α, peroxisome proliferator-activated receptor γ coactivator 1α; PRKDC, protein kinase, DNA-activated, catalytic polypeptide; RETNLB, resistin-like beta; ROC, receiver operating characteristic; TCGA, The Cancer Genome Atlas; TGFβ, transforming growth factor beta; WGCNA, weighted gene co-expression network analysis.
